# Influence of Polyethylene Terephthalate Powder on Hydration of Portland Cement

**DOI:** 10.3390/polym13152551

**Published:** 2021-07-31

**Authors:** Min Ook Kim, Jun Kil Park, Taek Hee Han, Joonho Seo, Solmoi Park

**Affiliations:** 1 Department of Civil Engineering, Seoul National University of Science and Technology, 232 Gongneung-ro, Nowon-gu, Seoul 01811, Korea; minookkim@seoultech.ac.kr; 2 Coastal Development and Ocean Energy Research Center, Korea Institute of Ocean Science and Technology, 385 Haeyang-ro, Yeongdo-gu, Busan 49111, Korea; jkpark@kiost.ac.kr (J.K.P.); taekheehan@kiost.ac.kr (T.H.H.); 3 Department of Civil and Environmental Engineering, Korea Advanced Institute of Science and Technology (KAIST), 291 Daehak-ro, Yuseong-gu, Daejeon 34141, Korea; junhoo11@kaist.ac.kr; 4 Department of Civil Engineering, Pukyong National University, 45 Yongso-ro, Nam-gu, Busan 48513, Korea

**Keywords:** polyethylene terephthalate powder, hydration, cement paste, exposure condition, material characterization, microstructural analysis

## Abstract

The management of plastic waste is a massive challenge and the recycling of plastics for newer applications is a potential solution. This study investigates the feasibility of using polyethylene terephthalate (PET) powder in cementitious composites. The changes in the strength and microstructure of Portland cement incorporating PET powder with different replacement ratios were systematically analyzed through the measurements of compressive strength, isothermal calorimetry, X-ray diffraction, thermogravimetric analysis, and Raman spectroscopy. In addition, the possible chemical changes of cement paste samples were studied upon exposure to different conditions, including deionized water, seawater, and simulated pore solution. Based on the test results and analysis, no apparent chemical changes were observed in the cement paste samples, regardless of the exposure conditions. In contrast, the PET powder incorporated into concrete exhibited remarkable changes, which may have occurred during the mixing process. The results also suggested that the maximum replacement ratio of PET powder should be less than 10% of the binder (by mass) to minimize its influence on cement hydration, due to the interaction between water and PET. The PET-containing samples showed the presence of calcium aluminate hydrates which were absent in the neat paste sample.

## 1. Introduction

The generation and disposal of plastic waste is a global concern. It is estimated that 99 million metric tons of plastic waste was generated in 2015, and the number is expected to triple to 155–265 million metric tons by 2060 [1]. Environmental problems related to the disposal of plastic waste have become more serious since China, which previously imported 45% of globally produced plastic waste, banned the import of most plastics a few years ago [2]. Single-use plastics make up the majority of the plastics produced and are directly related to the generation of plastic waste, and the demand for plastics is dramatically increasing in many areas, including the medical sector, owing to the outbreak of COVID-19 [3]. The issues of plastic waste are compounded by microplastic pollution in ecosystems [4]. Therefore, it is essential to create value-added applications for reused and recycled plastic products.

The packaging sector, which is a major plastic industry, uses approximately 40% of all plastic produced for single–use [5,6]. Thermoplastics, such as polyethylene, polyethylene terephthalate (PET), polypropylene, and polystyrene, are the most frequently used plastics in this sector [5,6]. Plastic recycling actively began many years ago and has been successful in employing the lifecycle of plastics at a commercial scale, with the help of regulations and policies. Traditionally, PET waste (i.e., beverage bottles) was recycled into fibers, whereas higher amounts could be embraced by the decontamination efficiencies with super-clean recycling processes [7,8,9,10]. This has expanded the application of PET waste to products in direct contact with food [7]. However, these approaches are not driven by cost reduction, but rather reliant on awareness of sustainable ethics by the respective business sectors [7].

The construction industry has been one of the largest consumers of recycled PET for many years [11,12,13]. The advantage of using recycled PET waste for construction materials is that it requires minimal pretreatment, which can often be costly, and therefore, a large volume of waste can be recycled at a relatively low cost [14]. Similar to the effect of incorporating any other fibers, PET fibers produced from PET bottle waste were reported to increase the elastic modulus of concrete by 16.9–21.5% when 0.25–0.75 vol.% PET was added [15]. A similar result was obtained by Kim et al. [16] where recycled PET fiber-reinforced concrete exhibited an increase in ductility and ultimate strength in comparison with unreinforced concrete. The use of recycled PET fibers also resulted in an increase in the compressive strength and transport properties of ultra–high performance concrete [17].

However, recycled PET does not always enhance the mechanical properties of concrete when recycled PET is added in a form other than fiber. In particular, a clear trend of decreasing strength with increasing PET waste content, which was added to replace fine or coarse aggregates, was observed in concrete [18,19,20,21]. On the one hand, the degradation of concrete strength with recycled PET somewhat limits the applications of recycled PET in construction; on the other hand, this has led to the diversification of the use of PET in concrete. For instance, the use of recycled PET in concrete can reduce the risk of spalling when exposed to fire [22,23] and improve the thermal insulation and energy efficiency of the building [24]. Therefore, the synergy of the improvement in thermal insulation and the reduction of the carbon footprint of buildings is achieved by the use of PET waste [24,25]. PET waste can also be used in controlled–low strength materials, which are designed to exhibit low strength (~3 MPa compressive strength). Despite the versatility of PET waste for use in concrete, the effect of PET on cement hydration has not been investigated yet. Understanding the influence of PET on the hydration of cement is required for designing concrete and should provide in-depth knowledge that enables the prediction of the overall performance of the final product.

There have been many attempts to recycle PET powder into a construction material, and previous studies have mostly investigated its mechanical properties and ways of utilizing such products (i.e., structural repair, lightweight concrete). However, the effect of PET on the hydration of Portland cement (PC) has not yet been systematically investigated. This study investigates the feasibility of using PET powder as a binder in cement material, which may be a potential application for recycled PET-type materials collected from marine environments. Specifically, the PET powder was incorporated as a binder for PC, and changes in strength, microstructure, and hydration were systematically investigated through the results of compressive strength tests, isothermal calorimetry, X-ray diffraction (XRD), Fourier transform infrared (FT-IR) spectroscopy, and Raman spectroscopy. Furthermore, the effects of different curing conditions, such as deionized water, seawater, and simulated pore solution, on the strength and microstructural changes were examined. From a hypothetical standpoint, an addition of PET to cement pastes could lead to an interaction between the PET, the cement grains and water and bring physicochemical changes that would otherwise not occur in a neat cement paste.

## 2. Materials and Methods

### 2.1. Materials and Sample Preparation

**Figure 1:**
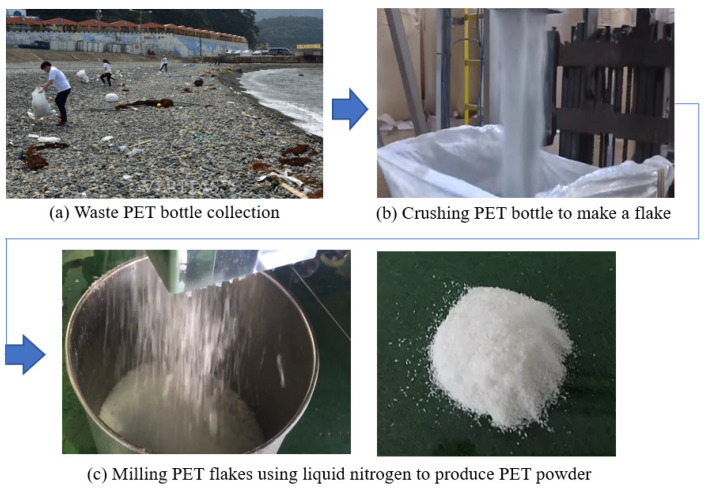
Preparation process of PET powder. (**a**) Waste PET bottle collection; (**b**) crushing PET bottle to make a flake; (**c**) Milling PET flakes using liquid nitrogen to produce PET powder.

The PET powder used in this study was produced by grinding PET bottles, as shown in Figure 1. The PET bottles were originally collected from Gamji Beach, Busan, South Korea. The collected PET bottles were first washed with a waterjet system to remove dust, salinity and marine organisms that may be present on the surface. The washed PET bottles were then fed into a crushing machine to produce PET flakes. The PET flakes were washed again with water before milling to produce PET powder. It should be noted that no chemicals were added in the washing water as they can influence the material properties of the PET. Liquid nitrogen was used to produce the PET powder, as shown in Figure 1. No selection or washing was performed during the production process. The measured specific gravity of the PET in this study was 1.26 ± 0.3, which is similar to the widely accepted range of 1.28–1.38 for the specific gravity for PET. The water absorptivity and porosity of PET were confirmed to be 0%, based on the experimental results. 

**Figure 2:**
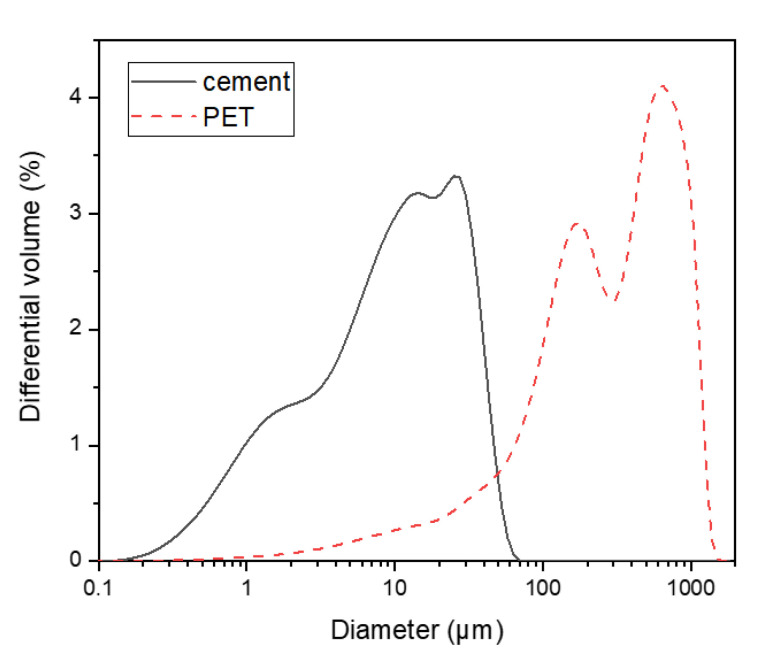
Particle size distributions of the cement and PET powder.

**Figure 3:**
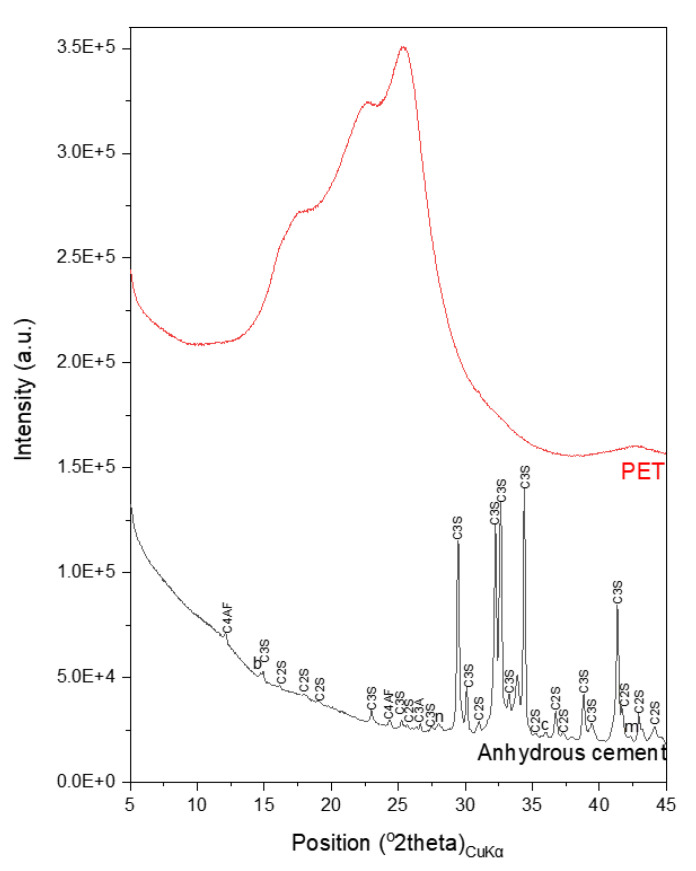
XRD patterns of the cement and the PET powder. Symbols are used to denote b: bassanite; c: calcite; m: periclase; n: thernadite.

The oxide composition of the Type I PC used in this study was obtained by X-ray fluorescence (XRF) analysis and is provided in Table 1. The XRD patterns and particle size distributions of the cement and the PET powder are shown in Figure 2 and Figure 3, respectively. The XRD pattern of the PET powder displayed amorphous humps, which are typically observed in milled PET [26].

For sample preparation, dry powders of cement and PET powder were mixed prior to adding deionized water. The PET/(cement + PET) fractions were 0%, 5%, 10% or 20% by mass, and the water/(cement + PET) fraction was 0.45 by mass. These mixture proportions were designed based on the preliminary test which determined the maximum amount of PET that can be added to the mixture. The mixtures were mixed for 5 min, and the fresh paste was cast into a mold. The samples were cured at room temperature for 60 days in sealed conditions, so that the samples reached a sufficient degree of hydration at the time of testing.

Another set of samples was prepared using PET powder and three different solutions. PET powder was immersed in deionized water, seawater, or a simulated pore solution of hydrated cement. These solutions were used to simulate the environment to which PET powder was likely to be exposed during service. The chemical composition of the seawater used in this study is summarized in Table 2. The simulated pore solution of hydrated cement was prepared according to the concentrations of ions, which were experimentally measured from hydrated cement samples, reported by Deschner et al. [27]. The powder-to-solution ratio was 1:100. The samples were stored at room temperature.

### 2.2. Test Methods

The compressive strengths of the prepared samples were measured according to displacement control at a constant rate of 1.0 mm/min. The hardened cement samples containing PET powder were crushed and ground into powder for characterization. Similarly, the PET powder samples immersed in different solutions were retrieved and vacuum filtered. The characterization techniques employed in this study include isothermal calorimetry, XRD, thermogravimetric analysis (TGA), FT–IR spectroscopy, and Raman spectroscopy.

Isothermal calorimetry was conducted using a three-point multipurpose conduction calorimeter (Tokyo–Riko Co., Ltd., Tokyo, Japan). Dry mixtures and water were mixed in the vessels, allowing the measurement of heat release at the start of mixing. XRD patterns were obtained using an X’Pert3–Powder X–ray diffractometer (PANalytical, Malvern, Worcestershire, UK), operating at 30 mA and 40 kV with Cu K-α radiation. The samples were scanned over a 2ϴ angle range of 5–65° with a step size of 0.026° for 1.5 h. TGA was conducted using DTG–60H (Shimadzu, Kyoto, Japan) at a heating rate of 10 °C/min in N_2_. FT–IR spectra were obtained using an FT–4100 spectrometer (JASCO, Tokyo, Japan). Raman spectra were obtained using an NRS–5100 Micro Raman Spectrometer (JASCO, Tokyo, Japan) with a 532-nm beam in the spectral range of 98–4000 cm^−1^.

## 3. Results and Discussion

### 3.1. Compressive Strength

**Figure 4:**
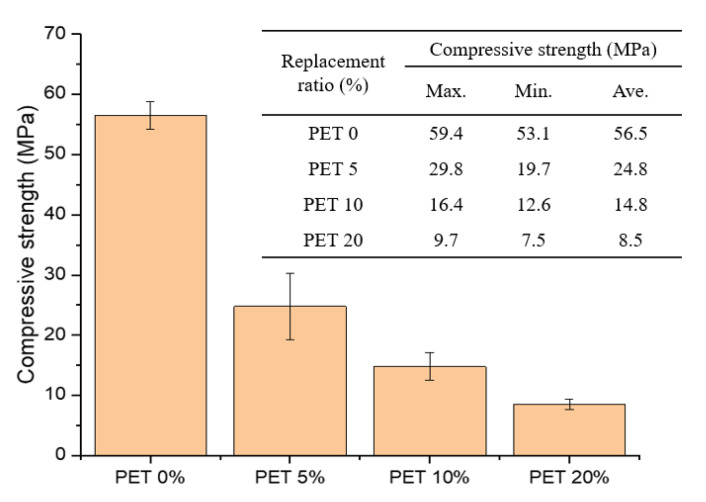
Average compressive strengths of cement paste samples with PET powder at different replacement ratios between 0 and 20%.

The average compressive strength values of the samples are shown in Figure 4. The average strengths of the samples incorporating 0%, 5%, 10%, and 20% PET powder were 56.5, 24.8, 14.8, and 8.5 MPa, respectively. As expected, the compressive strength showed a decreasing trend with increasing PET powder content. Specifically, the sample with 10% PET powder content exhibited a 74% reduced compressive strength compared to that of the control sample without PET.

These results are approximately twice as high as those of previous studies, which reported a 30–50% reduction in compressive strength with 10% PET content [28,29,30,31]. This significant reduction in compressive strength might be related to the types of PET material and/or prepared sample. Most previous studies used PET flakes rather than powder and produced concrete or cement mortar and not cement paste. A high PET powder content in cementitious materials can cause a significant reduction in compressive strength; thus, careful selection of the replacement ratio is important. The use of PET powder in cementitious materials can be advantageous for floating-type structures with reduced material density, but it should be carefully considered for structural applications.

### 3.2. Isothermal Calorimetry Results of Cement Paste Samples Incorporating PET Powder

**Figure 5:**
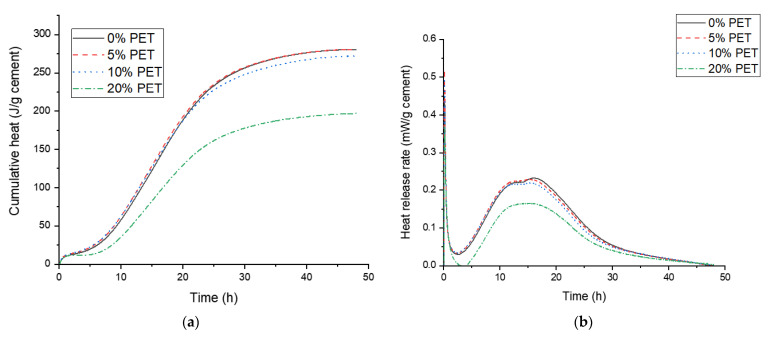
Isothermal calorimetry results of cement paste samples incorporating PET powder. (**a**) Cumulative heat and (**b**) heat release rate.

The isothermal calorimetry results for the cement pastes incorporating PET powder are shown in Figure 5. It was observed that the incorporation of up to 10% PET powder did not reduce the heat of hydration significantly, while there was a remarkable decrease in the cumulative heat released over 50 h when the PET content was increased to 20%. Thus, it is inferred that the extent of cement hydration can be affected by the amount of PET incorporated; an extremely high added amount can lead to a decrease in the extent of hydration. The reduced extent of hydration by the excessive addition of PET can be due to the hydrophilic site in the polar groups of PET that bind water molecules [32], therefore competing with cement grains which also require water. On the other hand, it is observed in Figure 5b that the positions of the silicate and alumina peaks at ~12 and 14 h [33], respectively, are consistent throughout the samples, suggesting that the dissolution of cement grains and formation of hydration products occur independent of PET incorporation. Considering that the silicate and alumina peaks are observed at similar positions for other samples, the reduced heat release in the sample incorporating 20% PET powder may be due to the high amount of PET acting as an insulator and causing an error in the heat measurement during calorimetry.

### 3.3. X-ray Diffraction Patterns of Cement Pastes with PET Powder

**Figure 6:**
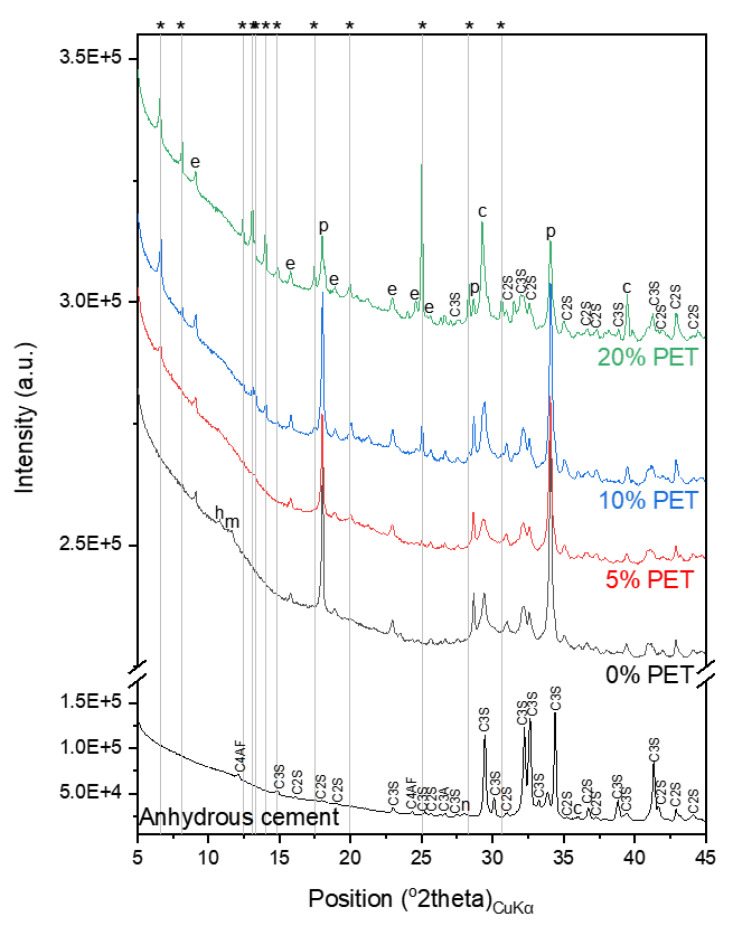
XRD patterns of cement paste samples incorporating PET powder. The following notations were used to denote: c: calcite; e: ettringite; h: hemicarboaluminate; m: monocarboaluminate; p: portlandite. Asterisks denote new phase identified in PET-incorporating samples.

The XRD patterns of the cement paste samples incorporating PET powder are shown in Figure 6. Typical hydration products of PC, such as portlandite, ettringite, hemicarboaluminate, and monocarboaluminate, were identified in the hydrated samples, thereby exhibiting no significant change in the hydration products due to PET powder incorporation.

**Figure 7:**
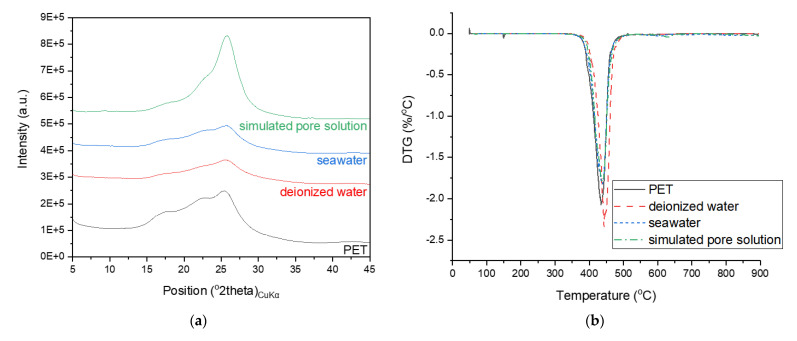

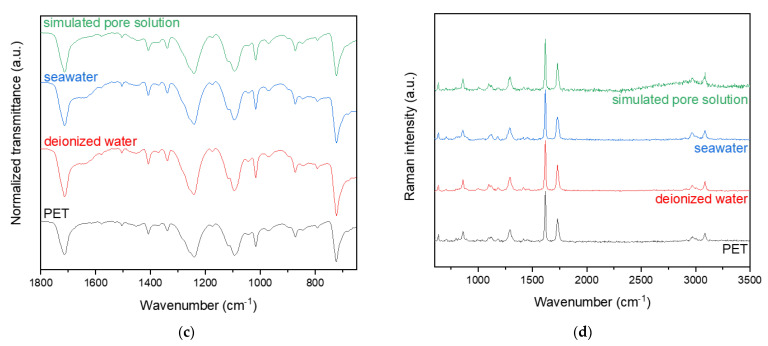
(**a**) XRD spectra, (**b**) DTG measurement, (**c**) FT–IR and (**d**) Raman spectra of neat PET powder and samples of PC with PET immersed in deionized water, seawater, or simulated pore solution.

The typical amorphous hump of the PET powder was absent in the XRD patterns of the hydrated samples incorporating PET powder. Instead, a new phase with some structural similarities to rubrene (a pattern matched with a report by Jurchescu et al. [34]) was identified, and this feature was particularly pronounced in the samples with a higher PET powder content. These peaks were attributed to alkaline terephthalates, whose formation has been reported in studies on PET fibers incorporated into cement-based materials owing to structural degradation under alkaline conditions [35,36,37]. Comparing these peaks with the XRD patterns of the PET powder immersed in deionized water, seawater, or simulated pore solution, as shown in Figure 7a, the formation of this new phase is observed to uniquely occur in the hydrated cement matrix. Although there are some differences in the intensities of the humps in the XRD patterns of PET powder immersed in different solutions, other characterization results (DTG, FT-IR, and Raman spectroscopy) in Figure 7 suggest that immersion in any of the three solutions does not necessarily drive such structural changes as those observed in the PET powder incorporated in hydrated PC.

### 3.4. Results from Thermogravimetric Analysis

**Figure 8:**
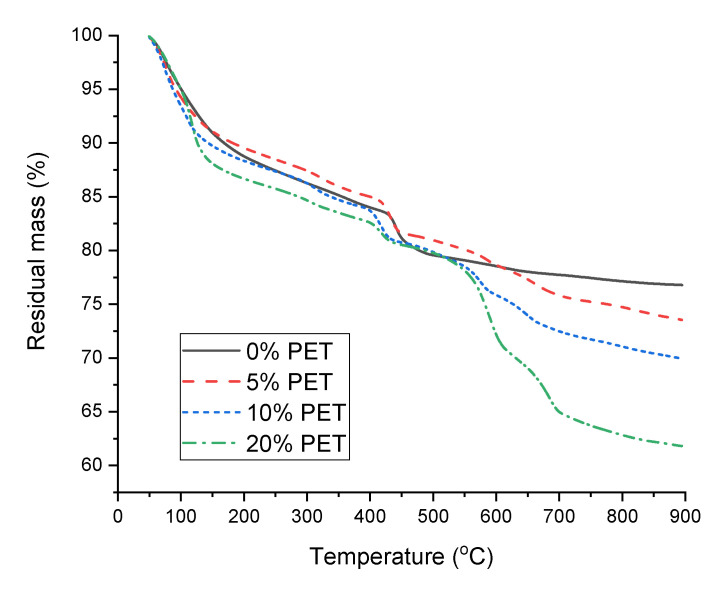

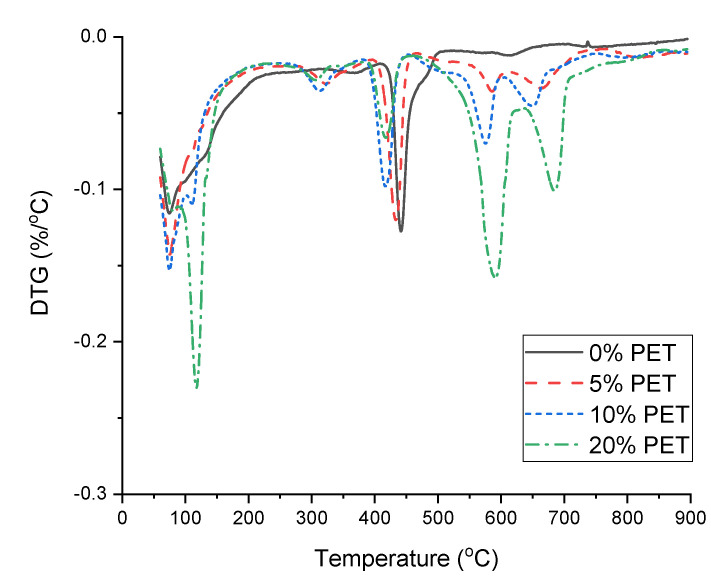
DTG curves of cement paste samples incorporating PET powder.

The DTG curves of the cement paste samples incorporating PET powder are shown in Figure 8, which provide quantitative information on the hydration products formed in the samples. The samples exhibited a notable weight loss at ~80 °C, owing to the evaporation of free water from the matrix [38]. In addition, the PET-incorporated samples exhibited another distinct weight loss at ~115 °C. As the magnitude of this weight loss dip increases with the PET content, this weight loss can be attributed to the evaporation of water from the PET−cement interfaces.

The shoulder at ~140 °C, which is attributed to the evaporation of water from AFm phases such as hemi/monocarboaluminate or monosulfoaluminate, was only visible for the 0% PET sample, while the other samples incorporating PET commonly showed a weight loss at ~315 °C, which can be attributed to the dehydration of calcium aluminum hydrates such as katoite or hydrogarnets containing silica [38]. Considering that PET addition does not seem to induce changes in hydration during the first 50 h, according to the isothermal calorimetry shown in Figure 5, it can be inferred from Figure 8 that the main role of PET is inducing the formation of calcium aluminate hydrates at later stages during the hydration of PC. This could be related to the reduced hydration degree as observed by the calorimetry in Figure 5, which could be associated with the interaction between water molecules and polar groups of PET [32].

All samples commonly showed a weight loss hump at 410–450 °C, whose position shifted to lower temperatures as the PET content increased. This weight loss is typical for hydrated PC and is attributed to the dehydroxylation of portlandite [38,39] and also coincides with that observed in the neat PET powder and PC samples immersed in different solutions (Figure 7b). Hence, this weight loss is also attributed to the degradation of cyclic oligomers and the release of acetaldehyde groups and anhydride oligomers from PET [40]. Under normal circumstances, quantifying this weight loss would allow the calculation of the amount of portlandite initially present in the samples, thereby providing a quantitative comparison of the hydration degrees of the samples. Unfortunately, the overlap of multiple phases in this weight loss region did not permit such an analysis.

All the PET-incorporating samples exhibited remarkable weight loss in the regions where decarbonation typically occurs. The absence of a carbonate peak of significant intensity in the XRD patterns of the PET–incorporating samples in Figure 6 implies that the decarbonation is associated with the interaction between PC, acetaldehyde groups, and anhydride oligomers produced by heating during the TGA analysis; thus, its occurrence is not anticipated under ambient conditions. A similar observation was reported in another study by Pereira et al. [41].

### 3.5. Results from Fourier–Transform Infrared Spectroscopy

**Figure 9:**
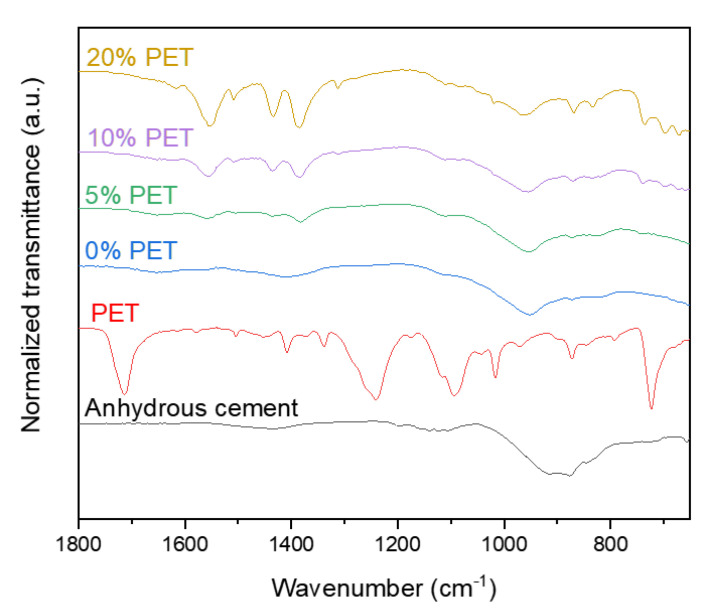
FT-IR spectra of samples incorporating PET powder.

The FT-IR spectra of the cement paste samples incorporating PET powder are shown in Figure 9. The FT–IR spectrum of the raw cement can be characterized by the wide and intense bands at 918 and 874 cm^−1^, which are attributed to the asymmetric stretching vibration of Si–O bonds in C_3_S [42]. This band shifted to ~950 cm^−1^ in the hydrated samples because of the Si-O stretching vibration, indicating the formation of C–S–H upon hydration of C_3_S and C_2_S [42,43]. The bands observed at 1715, 1241, 1089, 1018, 869, and 723 cm^−1^ in the FT-IR spectrum of the PET powder are attributed to the stretching of C=O in the carboxylic acid group, terephthalate group, and methylene group, and the vibration of ester C-O bond, aromatic rings, and benzene rings, respectively [44,45]. These bands were unchanged even after being in contact with deionized water, seawater, or simulated pore solution, as shown in Figure 7c, while none of these bands were present in the hydrated cement samples. The transmittance observed between 1350 and 1440 cm^−1^ in the spectra of the PET incorporating samples is attributed to the vibrational modes of the ethylene glycol segment [46,47], while the band at ~1550 cm^−1^ is associated with aromatic C–C stretching [48].

### 3.6. Results from Raman Spectroscopy

**Figure 10:**
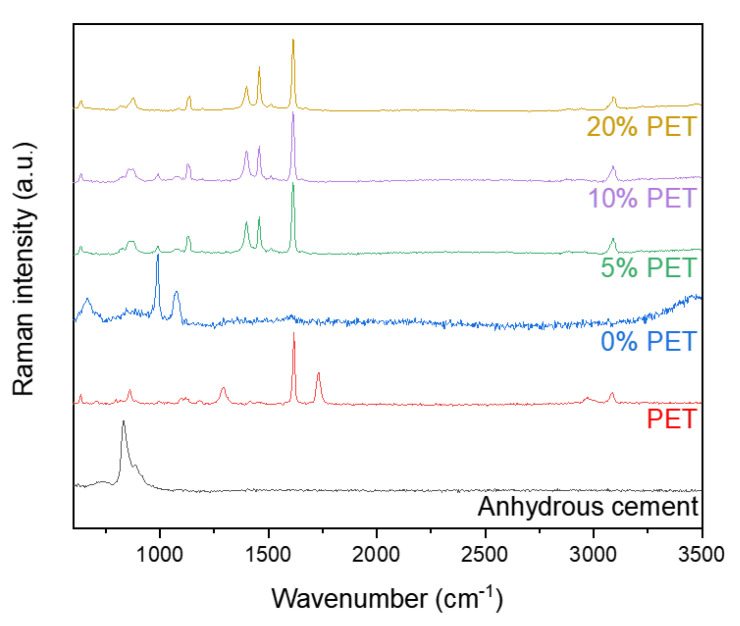
Raman spectra of cement paste samples incorporating PET powder.

The Raman spectra of the cement paste samples incorporating PET powder are shown in Figure 10. The Raman spectrum of raw cement showed an intense band at 835 cm^−1^ and a shoulder at 882 cm^−1^ due to the presence of C_3_S and C_2_S, respectively, while the peaks of monosulfate (986 cm^−1^) and ettringite (1077 cm^−1^) were observed in the spectrum of the hydrated cement sample [49].

PET powder can be characterized by the bands at 1617 and 1735 cm^−1^, which are associated with the ring mode 8a (in Wilson’s notation) and C=O stretching vibration [50,51]; similar to other results, the characteristics of PET seemed unchanged despite immersion in seawater or simulated pore solution. On the other hand, PET incorporation in cement led to structural alterations in the PET, as indicated by the bands at 1403 and 1454 cm^−1^, which are associated with CCH and CH_2_ bending, respectively [50,51]. The consistently strong band due to ring mode 8a in the spectra of the PET-incorporated samples indicates that partial structural alteration occurs in PET.

## 4. Conclusions

In this study, the effect of PET powder incorporation on the hydration of Portland cement was experimentally investigated. Different replacement ratios and exposure conditions were selected as the test variables. Based on the test results and comparisons with previous studies, the following conclusions can be drawn:

1. The hydration degree of PC can be influenced by a high PET fraction (e.g., 20%), as observed by the reduced heat released during the calorimetry, which can be associated with an interaction between water molecules and polar groups of PET.
2. Calcium aluminate hydrates such as katoite or hydrogarnets were formed in the samples containing PET powder (5–20%).
3. No clear microstructural and chemical changes were observed in the PET-incorporated PC samples exposed to different curing conditions, and the PET incorporated in cementitious material may not be affected by harsh environmental conditions.
4. Based on these observations, the PET-incorporated PC paste sample exhibited physical changes that might occur during the mixing rather than the hydration process.
5. The PET powder showed similar properties to rubrene, based on the XRD results, while the Raman spectrum showed different trends in the absence of C=O stretching vibration.

Existing literature states that waste plastics can be transformed into microplastics by physical impact. Upon immersing the PET-incorporated cement paste sample in seawater or cement pore solution, no significant structural change was observed in the original structure of the PET powder. Therefore, even if PET powder is incorporated into concrete, structural changes may not occur in harsh environments. The experimental results confirmed that when PET powder is applied to concrete, a change in the structure of some PET may occur, owing to the physical impact generated by the mixing process. In the case of concrete or cement grout, the pore structure of the matrix is complex and hermetically sealed, making it difficult to move the material. In the case of plastic waste, because microplastics are easily generated by physical impact, mixing and immobilizing plastic waste in concrete can reduce the generation of additional microplastics and can process a large amount of plastic waste in terms of value-added recycling.

## Figures and Tables

**Table 1:** Oxide composition (%) of the cement obtained by XRF spectroscopy.

CaO	SiO_2_	Al_2_O_3_	Fe_2_O_3_	SO_3_	MgO	K_2_O	TiO_2_	F	Na_2_O	ZnO	LOI *
64.50	17.30	4.36	3.70	3.25	2.51	1.07	0.30	0.17	0.16	0.13	2.16

* LOI: loss of ignition.

**Table 2:** Chemical composition of the seawater determined by ion chromatography.

Types of Ions	Cl^−^	Br^−^	SO_4_^2−^	Na^+^	K^+^	Ca^2+^	Mg^2+^
Concentration (mg/L)	21,075	51	2258	17,075	549	364	973

## Data Availability

Available upon request to the corresponding author.

## References

[1] Lebreton L., Andrady A. (2019). Future scenarios of global plastic waste generation and disposal. Palgrave Commun..

[2] Brooks A.L., Wang S., Jambeck J.R. (2018). The Chinese import ban and its impact on global plastic waste trade. Sci. Adv..

[3] Adyel T.M. (2020). Accumulation of plastic waste during COVID-19. Science.

[4] Rillig M.C., Lehmann A. (2020). Microplastic in terrestrial ecosystems. Science.

[5] Narancic T., O’Connor K.E. (2019). Plastic waste as a global challenge: Are biodegradable plastics the answer to the plastic waste problem?. Microbiology.

[6] Plastics Europe (2017). Plastics—The Facts 2017.

[7] Welle F. (2011). Twenty years of PET bottle to bottle recycling—An overview. Resour. Conserv. Recycl..

[8] Lonca G., Lesage P., Majeau-Bettez G., Bernard S., Margni M. (2020). Assessing scaling effects of circular economy strategies: A case study on plastic bottle closed-loop recycling in the USA PET market. Resour. Conserv. Recycl..

[9] Cruz S., Zanin M. (2006). PET recycling: Evaluation of the solid state polymerization process. J. Appl. Polym. Sci..

[10] Welle F. (2013). Is PET bottle-to-bottle recycling safe? Evaluation of post-consumer recycling processes according to the EFSA guidelines. Resour. Conserv. Recycl..

[11] Behera P., Noman M.T., Petrů M. (2020). Enhanced Mechanical Properties of Eucalyptus-Basalt-Based Hybrid-Reinforced Cement Composites. Polymers.

[12] Mahmood A., Noman M.T., Pechočiaková M., Amor N., Petrů M., Abdelkader M., Militký J., Sozcu S., Hassan S.Z.U. (2021). Geopolymers and Fiber-Reinforced Concrete Composites in Civil Engineering. Polymers.

[13] Noman M.T., Amor N., Petru M., Mahmood A., Kejzlar P. (2021). Photocatalytic Behaviour of Zinc Oxide Nanostructures on Surface Activation of Polymeric Fibres. Polymers.

[14] Siddique R., Khatib J., Kaur I. (2008). Use of recycled plastic in concrete: A review. Waste Manag..

[15] Bui N.K., Satomi T., Takahashi H. (2018). Recycling woven plastic sack waste and PET bottle waste as fiber in recycled aggregate concrete: An experimental study. Waste Manag..

[16] Kim S.B., Yi N.H., Kim H.Y., Kim J.-H.J., Song Y.-C. (2010). Material and structural performance evaluation of recycled PET fiber reinforced concrete. Cem. Concr. Compos..

[17] Alani A.H., Bunnori N.M., Noaman A.T., Majid T. (2019). Durability performance of a novel ultra-high-performance PET green concrete (UHPPGC). Constr. Build. Mater..

[18] Saikia N., de Brito J. (2014). Mechanical properties and abrasion behaviour of concrete containing shredded PET bottle waste as a partial substitution of natural aggregate. Constr. Build. Mater..

[19] Choi Y.-W., Moon D.-J., Chung J.-S., Cho S.-K. (2005). Effects of waste PET bottles aggregate on the properties of concrete. Cem. Concr. Res..

[20] Marzouk O.Y., Dheilly R., Queneudec M. (2007). Valorization of post-consumer waste plastic in cementitious concrete composites. Waste Manag..

[21] Mohammed A.A. (2017). Flexural behavior and analysis of reinforced concrete beams made of recycled PET waste concrete. Constr. Build. Mater..

[22] Nematzadeh M., Shahmansouri A.A., Fakoor M. (2020). Post-fire compressive strength of recycled PET aggregate concrete reinforced with steel fibers: Optimization and prediction via RSM and GEP. Constr. Build. Mater..

[23] Almeshal I., Tayeh B.A., Alyousef R., Alabduljabbar H., Mohamed A.M. (2020). Eco-friendly concrete containing recycled plastic as partial replacement for sand. J. Mater. Res. Technol..

[24] Maalouf C., Ingrao C., Scrucca F., Moussa T., Bourdot A., Tricase C., Presciutti A., Asdrubali F. (2018). An energy and carbon footprint assessment upon the usage of hemp-lime concrete and recycled-PET façades for office facilities in France and Italy. J. Clean. Prod..

[25] Belmokaddem M., Mahi A., Senhadji Y., Pekmezci B.Y. (2020). Mechanical and physical properties and morphology of concrete containing plastic waste as aggregate. Constr. Build. Mater..

[26] Font J., Muntasell J., Cesari E. (1999). Poly (butylene terephthalate) poly (ethylene terephthalate) mixtures formed by ball milling. Mater. Res. Bull..

[27] Deschner F., Lothenbach B., Winnefeld F., Neubauer J. (2013). Effect of temperature on the hydration of Portland cement blended with siliceous fly ash. Cem. Concr. Res..

[28] Ismail Z.Z., Al-Hashmi E.A. (2008). Use of waste plastic in concrete mixture as aggregate replacement. Waste Manag..

[29] Soroushian P., Plasencia J., Ravanbakhsh S. (2003). Assessment of reinforcing effects of recycled plastic and paper in concrete. Mater. J..

[30] Silva R.V., de Brito J., Saikia N. (2013). Influence of curing conditions on the durability-related performance of concrete made with selected plastic waste aggregates. Cem. Concr. Compos..

[31] Panyakapo P., Panyakapo M. (2008). Reuse of thermosetting plastic waste for lightweight concrete. Waste Manag..

[32] Merdas I., Thominette F., Tcharkhtchi A., Verdu J. (2002). Factors governing water absorption by composite matrices. Compos. Sci. Technol..

[33] Scrivener K., Martirena F., Bishnoi S., Maity S. (2018). Calcined clay limestone cements (LC3). Cem. Concr. Res..

[34] Jurchescu O.D., Meetsma A., Palstra T.T. (2006). Low-temperature structure of rubrene single crystals grown by vapor transport. Acta Crystallogr. Sect. B Struct. Sci..

[35] Silva D., Betioli A., Gleize P., Roman H., Gomez L., Ribeiro J. (2005). Degradation of recycled PET fibers in Portland cement-based materials. Cem. Concr. Res..

[36] Corinaldesi V., Nardinocchi A. (2016). Influence of type of fibers on the properties of high performance cement-based composites. Constr. Build. Mater..

[37] Das J., Halgeri A., Sahu V., Parikh P. (2007). Alkaline hydrolysis of poly (ethylene terephthalate) in presence of a phase transfer catalyst. Indian J. Chem. Technol..

[38] Lothenbach B., Durdzinski P., De Weerdt K. (2016). Thermogravimetric analysis. A Practical Guide to Microstructural Analysis of Cementitious Materials.

[39] Taylor H.F. (1997). Cement Chemistry.

[40] Samperi F., Puglisi C., Alicata R., Montaudo G. (2004). Thermal degradation of poly (ethylene terephthalate) at the processing temperature. Polym. Degrad. Stab..

[41] dos Santos Pereira A.P., da Silva M.H.P., Lima Júnior É.P., dos Santos Paula A., Tommasini F.J. (2017). Processing and characterization of PET composites reinforced with geopolymer concrete waste. Mater. Res..

[42] Del Bosque I.S., Martínez-Ramírez S., Blanco-Varela M.T. (2014). FTIR study of the effect of temperature and nanosilica on the nano structure of C–S–H gel formed by hydrating tricalcium silicate. Constr. Build. Mater..

[43] Bensted J., Varma S.P. (1974). Some applications of infrared and Raman spectroscopy in cement chemistry. Part 3-hydration of Portland cement and its constituents. Cem. Technol..

[44] Edge M., Wiles R., Allen N., McDonald W., Mortlock S. (1996). Characterisation of the species responsible for yellowing in melt degraded aromatic polyesters—I: Yellowing of poly (ethylene terephthalate). Polym. Degrad. Stab..

[45] Silverstein R.M., Bassler G.C. (1962). Spectrometric identification of organic compounds. J. Chem. Educ..

[46] Miyake A. (1959). The infrared spectrum of polyethylene terephthalate. I The effect of crystallization. J. Polym. Sci..

[47] Schmidt P. (1963). Polyethylene terephthalate structural studies. J. Polym. Sci. Part A Gen. Pap..

[48] Vijayakumar S., Rajakumar P. (2012). Infrared spectral analysis of waste pet samples. Int. Lett. Chem. Phys. Astron..

[49] Bensted J. (1976). Uses of Raman spectroscopy in cement chemistry. J. Am. Ceram. Soc..

[50] Boerio F., Bahl S., McGraw G. (1976). Vibrational analysis of polyethylene terephthalate and its deuterated derivatives. J. Polym. Sci. Polym. Phys. Ed..

[51] Rebollar E., Pérez S., Hernández M., Domingo C., Martín M., Ezquerra T.A., García-Ruiz J.P., Castillejo M. (2014). Physicochemical modifications accompanying UV laser induced surface structures on poly (ethylene terephthalate) and their effect on adhesion of mesenchymal cells. Phys. Chem. Chem. Phys..

